# Antifungal Peptide SP1 Damages Polysaccharide Capsule of Cryptococcus neoformans and Enhances Phagocytosis of Macrophages

**DOI:** 10.1128/spectrum.04562-22

**Published:** 2023-03-14

**Authors:** Yan Liu, Yang Zhang, Xi Zhao, Weilai Lu, Yuxin Zhong, Yu V. Fu

**Affiliations:** a State Key Laboratory of Microbial Resources, Institute of Microbiology, Chinese Academy of Sciences, Beijing, China; b Savaid Medical School, University of Chinese Academy of Sciences, Beijing, China; c School of Life Sciences, Division of Life Sciences and Medicine, University of Science and Technology of China, Hefei, China; d Department of Pancreatic and Gastric Surgery, National Cancer Center/National Clinical Research Center for Cancer/Cancer Hospital, Chinese Academy of Medical Sciences and Peking Union Medical College, Beijing, China; University of Debrecen

**Keywords:** antifungal peptide, *Cryptococcus neoformans*, capsule, phagocytosis of macrophage, biofilm

## Abstract

Cryptococcus neoformans is a fungal pathogen which causes nearly half a million deaths worldwide each year. Under host-relevant conditions, it produces a characteristic polysaccharide capsule. The polysaccharide capsule is one of the main virulence factors of C. neoformans, which involves antiphagocytosis and immune responses of the host to cause a lack of an immune. Meanwhile, the polysaccharide capsule is a promising drug target because of the absence of analogs in the host. Here, we demonstrate that antifungal peptide SP1, which is derived from the N terminus of Saccharomyces cerevisiae GAPDH (glyceraldehyde-3-phosphate dehydrogenase), disrupts the polysaccharide capsule of C. neoformans H99. The mechanism is possibly due to the interaction of SP1 with glucuronoxylomannan (GXM). Disruption of the polysaccharide capsule enhances the adhesion and phagocytosis of C. neoformans H99 by macrophages and reduces the replication of C. neoformans H99 within macrophages. Additionally, SP1 exhibits antifungal activity against cryptococcal biofilms associated with the capsular polysaccharides. These findings suggest the potential of SP1 as a drug candidate for the treatment of cryptococcosis.

**IMPORTANCE** C. neoformans is an opportunistic pathogen that causes invasive infections with a high mortality rate. Currently, the clinical drugs available for the treatment of cryptococcosis are limited to amphotericin B, azoles, and flucytosine. Amphotericin is nephrotoxic, and the widespread use of azoles and 5-flucytosine has led to a rapid development of drug resistance in C. neoformans. There is an urgent need to develop new and effective anticryptococcal drugs. Targeting virulence factors is a novel strategy for developing antifungal drugs. The antifungal peptide SP1 is capable of disrupting the polysaccharide capsule, which is a principal virulence factor of C. neoformans. Studying the mechanism by which SP1 damages the polysaccharide capsule and investigating the potential benefits of SP1 in removing C. neoformans from the host provides baseline data to develop a therapeutic strategy against refractory cryptococcal infections. This strategy would involve both inhibiting virulence factors and directly killing C. neoformans cells.

## INTRODUCTION

Fungal infections are a major concern for human health and are estimated to kill 150 people every hour globally (1, 2). Cryptococcus neoformans is the main clinical pathogen in systemic mycosis (3, 4). Cryptococcosis is one of the deadliest diseases caused by fungal infection (5). C. neoformans can infected many people possibly in a form that survives through latency in the lung without any symptoms (6, 7). However, for immunocompromised individuals, especially those with AIDS, infection leads to cryptococcal pneumonia and fatal cryptococcal meningitis (8, 9). According to the latest surveys, C. neoformans infects at least 278,000 people worldwide annually and results in more than 181,000 deaths (10). If patients infected with C. neoformans are not treated in time, the mortality rate can be 100% (11). The common therapeutic protocol for cryptococcosis includes the antifungals amphotericin B, flucytosine, and fluconazole (12). However, owing to the side effects and increase in drug-resistant strains, cryptococcal infections are becoming more and more serious (11). Additionally, in the past 25 years, no new antifungal drugs have been approved by the Food and Drug Administration for clinical use (13). Therefore, there is an urgent need for new and effective antifungal drugs.

The main virulence factor of C. neoformans is the polysaccharide capsule wrapped around the cell wall, and this plays a key role in the successful colonization and pathogenesis in the host (14, 15). The weak immunogenicity of polysaccharide capsules limits the ability of macrophages to phagocytose fungal cells (16). If the encapsulated cells are phagocytosed, the polysaccharide capsules can continue to protect cells from macrophage killing by providing resistance to reactive oxygen species released by macrophages (17, 18). The phagocytosed C. neoformans can replicate in macrophages, escaping from the lungs through the Trojan horse mechanism (19). C. neoformans then breaks through the blood-brain barrier to reach the brain, causing fatal meningitis (20). Furthermore, C. neoformans releases a large amount of the polysaccharide capsule component glucuronoxylomannan (GXM) during infection, leading to a harmful effect on the host immune response (21, 22). GXM inhibits neutrophil migration, dendritic cell activation, and antigen presentation. GXM can induce T-cell apoptosis and stimulate the production of proinflammatory and anti-inflammatory cytokines (23–28). Destroying the polysaccharide capsule or blocking the release of GXM can promote the phagocytosis of macrophages to reduce the fungal burden in the organism. Hypocapsular and acapsular mutants can survive and reproduce *in vitro* but display reduced or no virulence in a mouse model of infection (29).

C. neoformans can easily to form biofilm, and the polysaccharide capsules are necessary for biofilm formation. Studies have shown that the acapsular mutant *cap59*Δ cannot form biofilms (30). Biofilms on medical devices (such as ventricular atrial shunt conduits, artificial valves, and prostheses) are resistant to antifungal therapy and can cause serious health problems (31). Given the perniciousness of the capsular polysaccharide during the infection, drugs that both weaken the virulence of capsular polysaccharide and inhibit growth or kill cells would be desirable to reduce the burden of fungi in susceptible patients. Moreover, as the capsular polysaccharide or an analogue does not exist in the healthy host, the capsular polysaccharide is an ideal drug target.

SP1, an antifungal peptide derived from Saccharomyces cerevisiae GAPDH, has a specific antifungal effect against Cryptococcus (32, 33). Interestingly, we observed that treatment with SP1 specifically damaged the capsule of C. neoformans (32). In this study, we investigated the possible mechanism for the capsule damage caused by SP1, providing a basis for a treatment strategy to control refractory fungal infections by inhibiting virulence factors.

## RESULTS

### Treatment of SP1 causes a flocculation-like phenotype of C. neoformans H99.

**FIG 1:**
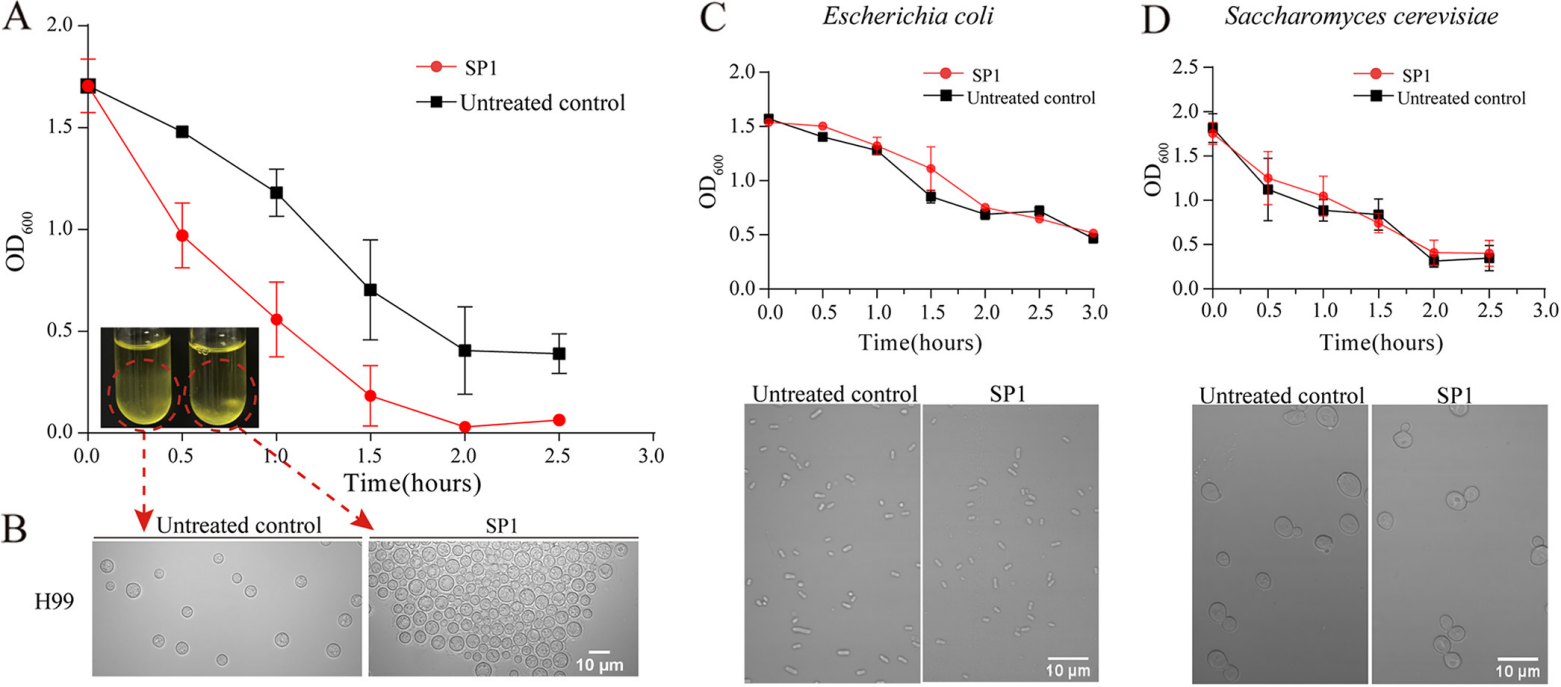
SP1 confers the flocculation-like phenotype in C. neoformans H99. (A) Sedimentation of C. neoformans H99 with or without SP1 treatment. The cell concentration in the upper stagnate culture (OD_600_) was measured brief vortexing at each 30-min time points up to 2.5 h. (B) C. neoformans H99 cells clump together after SP1 treatment. Cells from the bottom of the tube were examined with a microscope. (C) No flocculation of Escherichia coli was observed after SP1 treatment. (D) No flocculation of Saccharomyces cerevisiae was observed after SP1 treatment. The cells without SP1 treatment were used as the control group.

Previously, we reported that SP1 can specifically kill C. neoformans and Cryptococcus gatti (32). Scanning electronic microscopy showed that SP1 damaged the capsule of C. neoformans H99 (32). When we treated C. neoformans strain H99 with 8 μM SP1, we noticed that the treatment caused a flocculation-like phenotype (Fig. 1A and B), and treatment with 8 μM SP1 for 30 min was sufficient to trigger this. Increasing the concentration of SP1 gradually shortened the flocculation time to 2 min if treated with 128 μM SP1. The times required for flocculation to occur were 30 min (8 μM SP1), 10 to 20 min (16 μM SP1), 10 min (32 μM SP1), 2 to 3 min (64 μM SP1), and 1 to 2 min (128 μM SP1). At 4 μM SP1, no flocculation of C. neoformans H99 was observed. However, flocculation was not observed with Escherichia coli or Saccharomyces cerevisiae when treated with the same concentration or even a higher concentration (256 μM) of SP1 (Fig. 1C and D).

**FIG 2:**
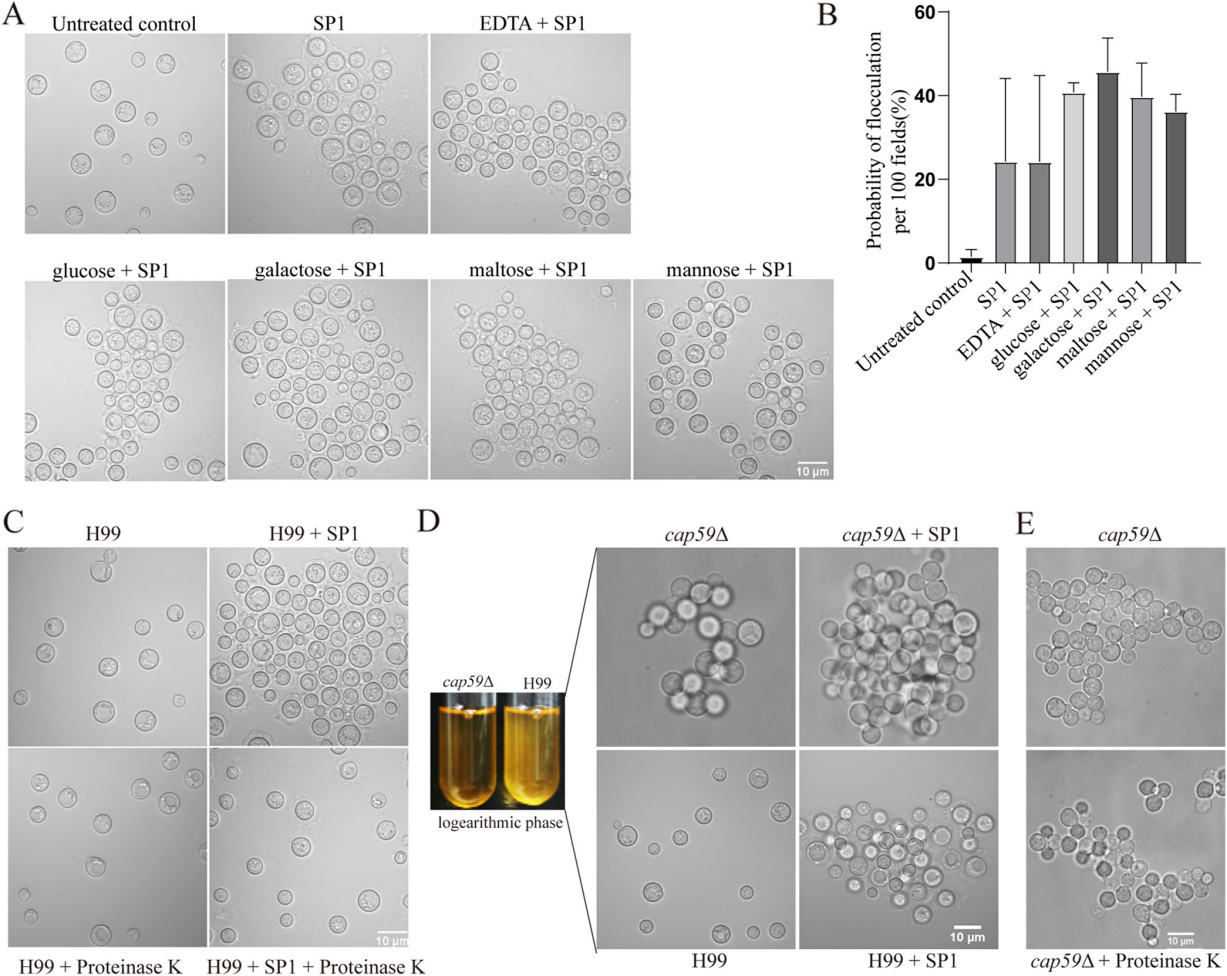
Mechanism of SP1-induced flocculation in C. neoformans H99. (A) Effects of carbohydrates and EDTA on the flocculation of C. neoformans H99 induced by SP1. After adding 5 mM EDTA, 20 mM mannose, 20 mM maltose, 20 mM galactose, or 20 mM glucose to C. neoformans H99 cultures, the cell cultures were treated with 8 μM SP1 for 30 min. Flocculation was observed through a microscope. Cells without any treatment were set as the negative control, and cells treated only with SP1 were the positive control. (B) Flocculation in 100 randomly selected visual fields was analyzed under the indicated experimental conditions. Student’s *t* test was used to evaluate the significance between data sets, and a *P* value of <0.05 (*) indicates a significant difference between the two data sets. (C) The treatment of proteinase K dispersed SP1-induced cell aggregation of C. neoformans H99. After flocculation was induced with SP1, 3 mg/mL proteinase K was added. Images were taken after 30 min of treatment. (D) Flocculation of acapsular mutant *cap59*Δ with or without 8 μM SP1. (E) Cell aggregation that formed spontaneously in the *cap59*Δ mutant was not dispersed by the treatment of protease K.

Given that the virulence of C. neoformans H99 decreased with the increase of the degree of flocculation (34), we investigated the possible mechanism of flocculation caused by SP1. For C. neoformans H99, treatment with 8 μM SP1 for 30 min killed only 11% of the cells (see Fig. S1 in the supplemental material) with obvious flocculation (Fig. 2B), although we did not observe any cell debris after treatment. Thus, it is unlikely that the flocculation was due to the aggregation of cellular substances released from the cells killed by SP1. Previous studies have shown that the lung surfactant protein SP-D can bind to the carbohydrates of the cell wall and induce the aggregation of acapsular C. neoformans in the presence of calcium ions (35, 36). We performed EDTA and carbohydrate competition experiments to verify whether SP1 triggered C. neoformans H99 flocculation by the same mechanism. As shown in Fig. 2A and B, the presence of EDTA, mannose, galactose, maltose, or glucose did not inhibit the SP1-induced flocculation of C. neoformans H99 cells (*P > *0.05). This observation suggested that SP1 caused flocculation by a different mechanism from that of SP-D.

The transient flocculation that occurs during the cultivation of C. neoformans is easily dispersed by vortexing (34). However, the aggregation of H99 cells caused by SP1 was not reversed by vigorous vortexing (Fig. S2). We observed that the acapsular *cap59*Δ mutants naturally flocculated in the logarithmic phase of culturing and that the degree of aggregation was increased after 8 μM SP1 was added to the culture of the *cap59*Δ mutant (Fig. 2D). Similarly, the cell aggregation of the *cap59*Δ mutant without SP1 or in the presence of SP1 was resistant to vortexing (Fig. 2D and Fig. S4). Combining these observations of the *cap59*Δ mutants and the fact that the dense network structure of polysaccharide capsule was severely damaged after treatment of SP1 (32), we postulated that SP1 destroyed the capsule to expose proteins or polysaccharide, and the interactions of these exposed substances might induce the flocculation of C. neoformans H99. When the flocs were treated with 3 mg/mL proteinase K, the cell aggregation caused by SP1 was dispersed (Fig. 2C). In contrast, proteinase K digestion did not affect the spontaneous cell aggregation of the *cap59*Δ mutant (Fig. 2E). These observations implied that the SP1-induced flocculation of C. neoformans H99 might be due to the interactions mediated by SP1 or the exposed proteins. The mechanism is also different from the mechanism of spontaneous flocculation in *cap59*Δ mutants that might be mediated by polysaccharide.

### SP1 interacts with the glucuronoxylomannan of polysaccharide capsule.

As SP1 damaged the polysaccharide capsule in a very short time, we hypothesized that SP1 would directly bind and break the polysaccharide capsule rather than regulating the expression of genes involved in capsule synthesis. Furthermore, our previous study showed that the killing activity of SP1 on C. neoformans H99 was due to an apoptosis-like cell death rather than direct damage to the capsule. SP1 does not form pores on the cell membrane of C. neoformans H99 but interacts with membrane ergosterol and enters the vacuole to induce apoptosis-like cell death (32). Theoretically, the capsule can prevent SP1 from reaching the cell membrane or inside of C. neoformans H99, and the MIC of SP1 against C. neoformans H99 would change if damage to the capsule caused by SP1 was altered. If SP1 hardly damaged the capsule of a C. neoformans H99 mutant, the intact capsule would prevent the access of SP1 to the cell membrane. Under this condition, we expected that the MIC would increase. Therefore, we examined the MIC of SP1 against a series of capsular defect mutants to determine the possible interacting targets of SP1 in the capsule (Table 1). The capsule of C. neoformans is composed primarily of two types of polysaccharides, GXM and glucuronoxylomannogalactan (GXMGal) (37, 38). Both GXM and GXMGal contain xylose, glucuronic acid, and *O*-acetyl modifications, and *O*-acetyl modification mainly occurs on GXM (39). As shown in Table 1, the MIC of SP1 against wild-type C. neoformans H99 cells was 8 μM, which was same as that of SP1 against GXMGal-deficient strains [(*uge1*Δ [CNAG_00697] and *uge1*Δ [CNAG_03096])] and 2-fold higher than that of the xylose-deficient mutant *uxs1*Δ. The MIC of SP1 against *O*-acetyl-deficient mutant *cas1*Δ was increased by 7-fold compared with that of the wild-type cells (Table 1). These results implied that oxyacetylation modification would be related to SP1 interaction with capsule.

**TABLE 1:**
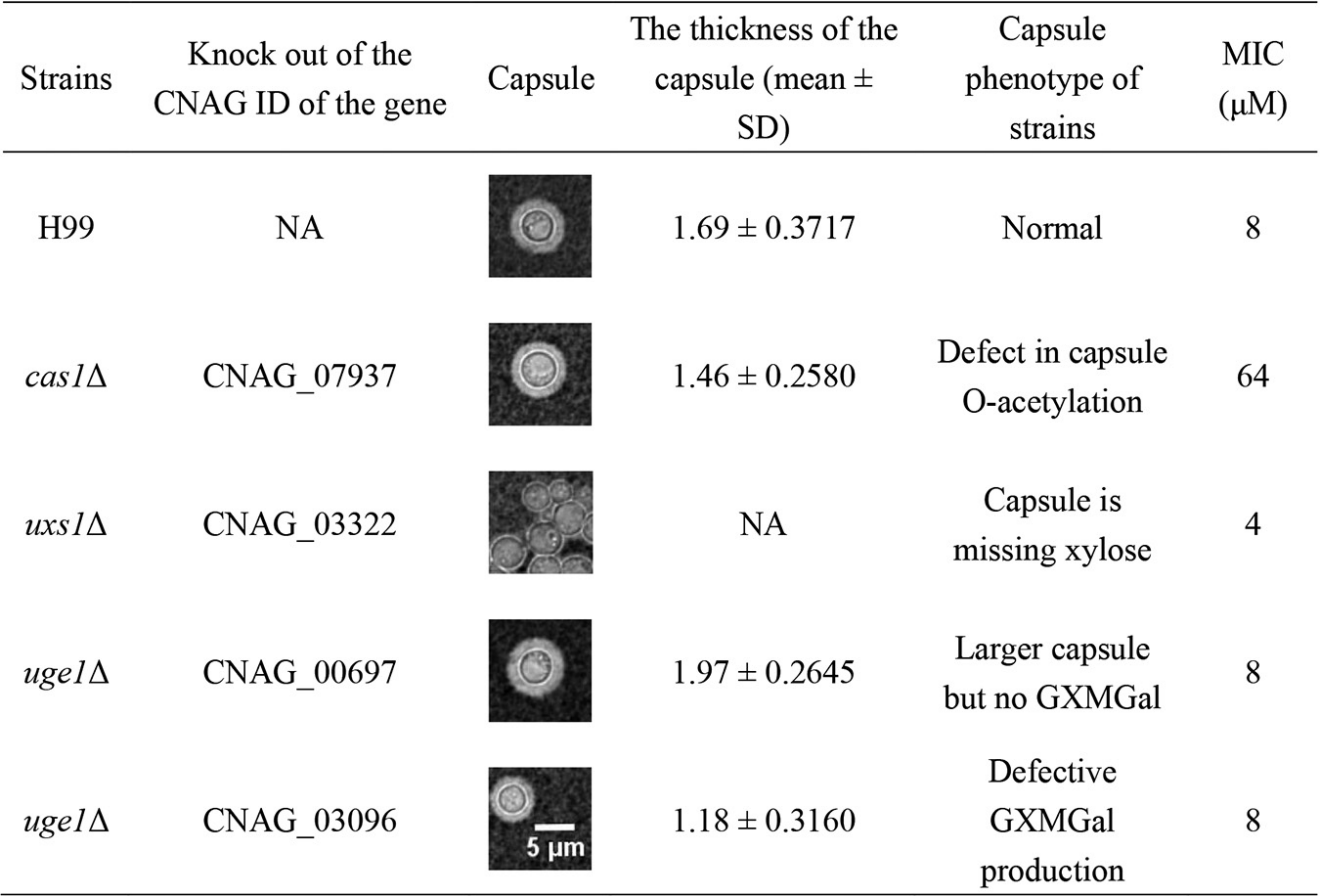
MIC of SP1 against polysaccharide capsule mutant strains^*a*^

a NA indicates that the gene is not knocked out or the thickness of the capsule cannot be measured because the capsule is too thin.

**FIG 3:**
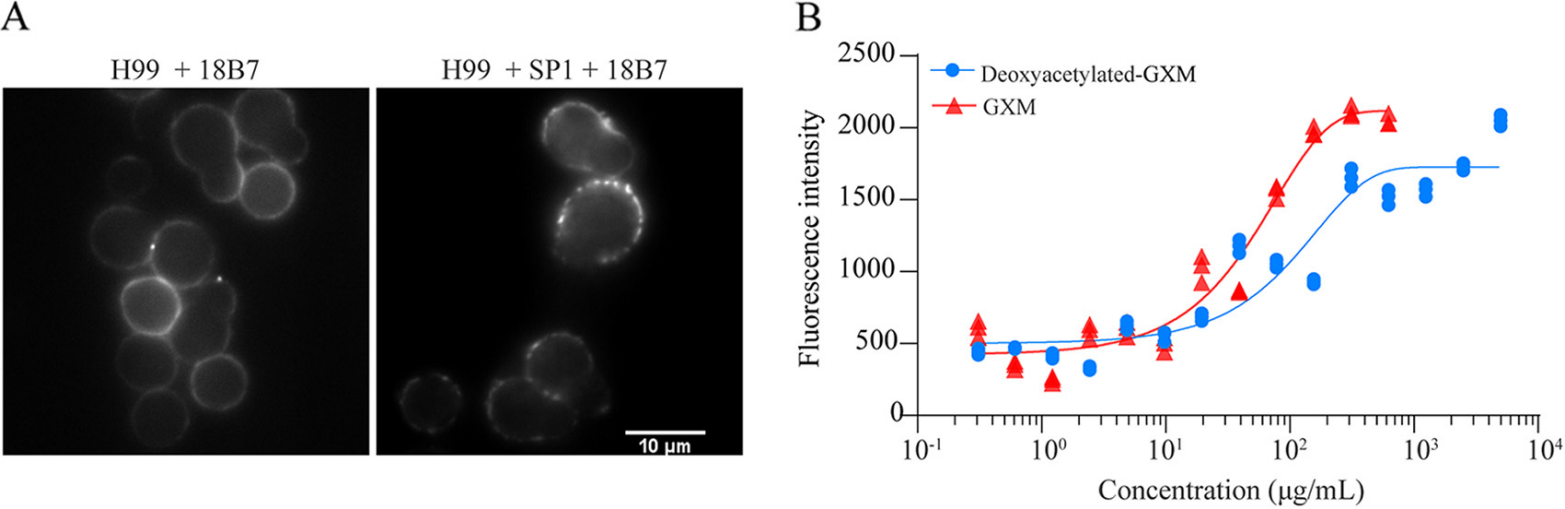
SP1 binds to GXM of the polysaccharide capsule. (A) Immunofluorescent images of C. neoformans H99 cells with or without incubation with SP1; the primary antibody and the secondary antibody were 18B7 and goat anti-mouse IgG conjugate (H+L) Qdot 655, respectively. (B) MST assay for interaction between GXM/deoxyacetylated GXM and SP1-FITC.

Furthermore, using the polysaccharide capsule monoclonal antibody 18B7 (MAb 18B7) against GXM oxyacetylation, we performed an immunofluorescence competition experiment to examine whether SP1 interacted with GXM. As shown in Fig. 3A, fluorescent images of control-peptide-treated cells displayed an annular pattern, whereas SP1-treated cells showed a punctate pattern when incubated with fluorescent MAb 18B7. These results indicated that the *O*-acetyl modification on GXM would be a potential binding site for SP1. There is another possibility that SP1 breaks the capsule to form a punctate structure. To obtain independent evidence for the binding of SP1 to GXM, we used a microscale thermophoresis (MST) assay to detect the binding ability of SP1-fluorescein isothiocyanate (FITC) with GXM or deoxyacetylated GXM. As shown in Fig. 3, there is an evident binding between SP1 and GXM with a dissociation constant (*K_d_*) of 4.13 ± 0.857. The *K_d_* value for the interaction between SP1 and deoxyacetylated GXM was 107 ± 15.8. Collectively, these observations suggested that SP1 interacted with GXM and that the oxyacetylation modification of GXM played an important role during the interaction.

**FIG 4:**
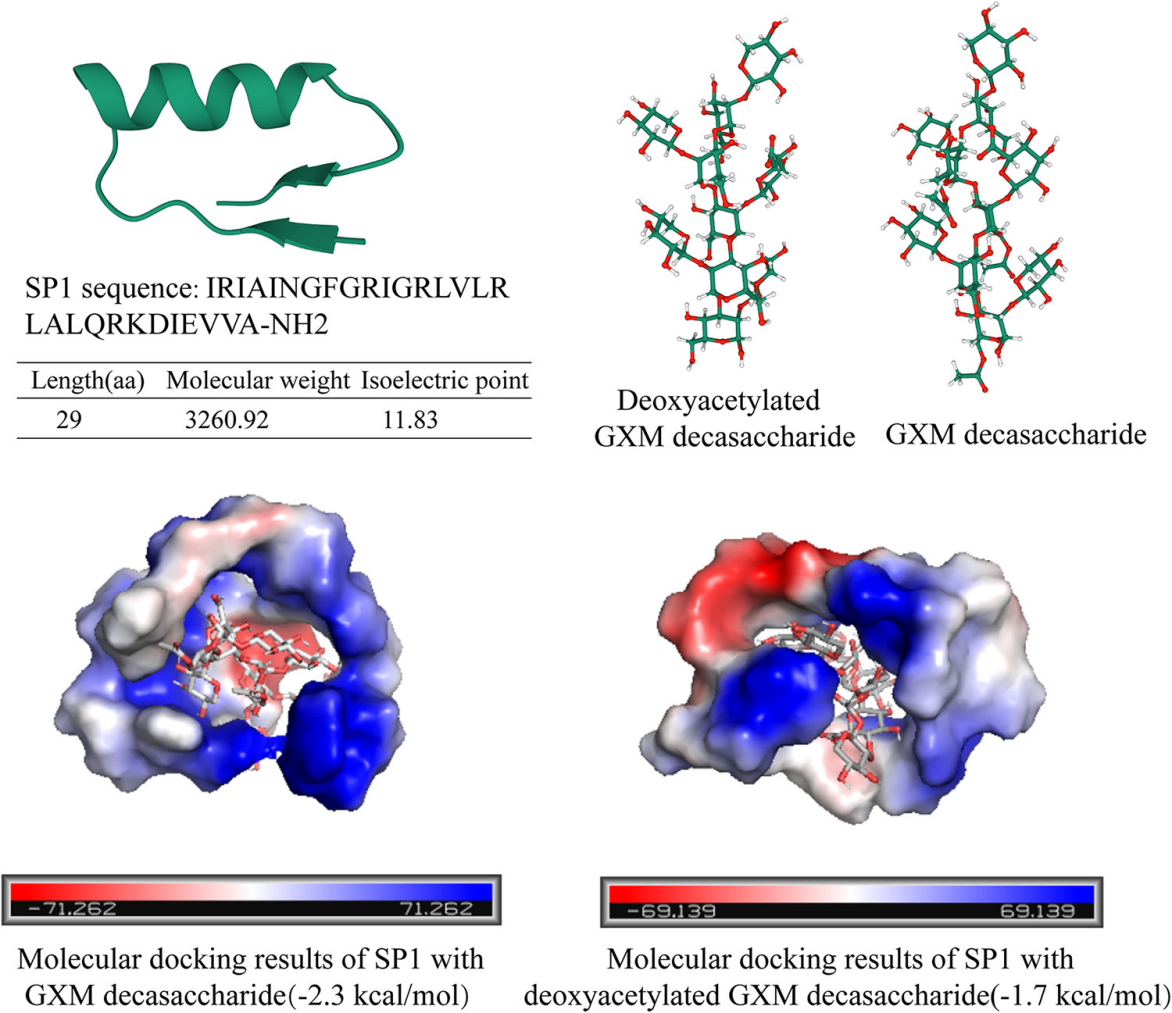
Molecular docking results of SP1 and GXM. (A) The structure of SP1 predicted by AlphaFold2. (B) The structure of GXM decasaccharide without (left) or with (right) oxyacetylation predicted by the GLYCAM carbohydrate builder. (C) Molecular docking results of SP1 with GXM (left) or GXM without oxyacetylation (right).

To determine how the oxyacetylation modification affects the interaction between SP1 and GXM, we analyzed the molecular docking of SP1 and GXM using AutoDock Vina and PDBePISA (https://www.ebi.ac.uk/msd-srv/prot_int/pistart.html). The structure of SP1 was predicted using AlphaFold2 (Fig. 4A), and the structure of GXM decasaccharide with or without oxyacetylation was predicted using the GLYCAM carbohydrate builder (Fig. 4B). These analyses showed that the amino acids involved in the interaction between SP1 and GXM were polar amino acids such as arginine (Arg), isoleucine (Ile), asparagine (Asn), aspartic acid (Asp), and glutamic acid (Glu). If GXM decasaccharide was deoxyacetylated, Asp became the only amino acid that was involved in the interaction (Fig. S3). The results suggested that the GXM decasaccharide formed hydrogen bonds with the Asn. However, when the GXM decasaccharide was deoxyacetylated, no hydrogen bond was formed in the interaction between SP1 and GXM decasaccharide (Fig. S3). Thus, the predicted binding affinity decreased from −2.3 to −1.7 kcal/mol (Fig. 4C).

### SP1 treatment inhibits the GXM release and upregulates expression of genes related to polysaccharide capsule synthesis.

**FIG 5:**
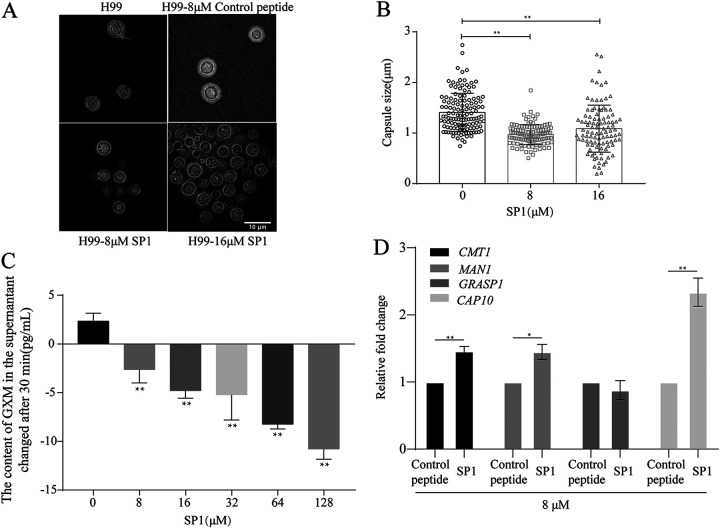
The reduction of GXM content in the supernatant by SP1 results in upregulated expression of capsule-related genes. (A) Representative India ink images showing the capsule of C. neoformans H99 after 30 min of SP1 treatment; the polysaccharide capsule is indicated by a red arrow. (B) Capsule size measurements of the control and SP1-treated C. neoformans H99. The capsule size of 100 cells was measured in each group; each circle, square, or triangle represents one C. neoformans H99 cell. (C) Content of GXM in the supernatant after treating C. neoformans H99 cells with SP1. (D) Relative fold changes of the expression of capsule-related genes after SP1 treatment; the GAPDH gene was used as a reference. Error bars represent the standard deviation (SD) of triple replicates. Asterisks represent the *P* value significance (**, *P* < 0.01; *, *P* < 0.05) calculated using one-way analysis of variance (ANOVA).

To investigate how SP1 destroys the capsules through interaction with GXM, we first used India ink negative staining to examine the effect of SP1 on the size of the capsule. The SP1-treated cells had significantly smaller polysaccharide capsules than untreated cells (Fig. 5A and B; *P < *0.01). To verify whether SP1 shed GXM from the polysaccharide capsule to form a smaller capsule, we used an enzyme-linked immunosorbent assay (ELISA) to detect the content of GXM in the supernatant of cell cultures after treating the H99 cells with different concentrations of SP1. As shown in Fig. 5C, the GXM content in the supernatant of C. neoformans H99 cultures gradually decreased with the increase of SP1 concentration used in the treatment. One explanation of this observation would be that the interaction between SP1 and GXM affected the antibody binding to GXM in the ELISA. To rule out this possibility, we collected the supernatant from the C. neoformans H99 culture after this reached the logarithmic phase of growth. The supernatant was then incubated with different concentrations of SP1, followed by the quantification of GXM by ELISA. The GXM content in the supernatant of C. neoformans H99 culture incubated with SP1 was not significantly different from that of the supernatant without incubation with SP1 (Fig. S5), indicating that SP1 does not interfere with the measurement of GXM content in the ELISA. Therefore, SP1 prevents the release of GXM rather than causing GXM to be shed from the capsule during the damage to capsule. Moreover, we noticed that SP1 only significantly reduced the level of extracellular deoxyacetylated-GXM at a concentration of 64 μM (Fig. S6), which is 8-fold higher than that found in wild-type strains (8 μM). This observation supports the hypothesis that the oxyacetylation modification of GXM might be important for the binding of SP1.

Additionally, given that C. neoformans can sense the content of the extracellular capsule to regulate the synthesis and release of capsule components (40), we evaluated the expression levels of capsular-related genes (*CMT1*, *MAN1*, *GRASP1*, and *CAP10*) after treating C. neoformans H99 cells with SP1. SP1-treated cells demonstrated an ~1.5-fold upregulation of *CMT1* and *MAN1* expression compared with that in control-peptide-treated cells (Fig. 5D; *P < *0.05). Furthermore, *CAP10*, an essential capsular gene, displayed an ~2-fold increase in expression in SP1-treated fungi compared with that in control-peptide-treated cells (Fig. 5D; *P < *0.01). Collectively, our results indicate that SP1 interacted with GXM to cause serious damage to the capsular network structure, which decreased the extracellular GXM content and upregulated expression of capsule-related genes.

### SP1 exhibits antifungal activity against C. neoformans H99 biofilms.

**FIG 6:**
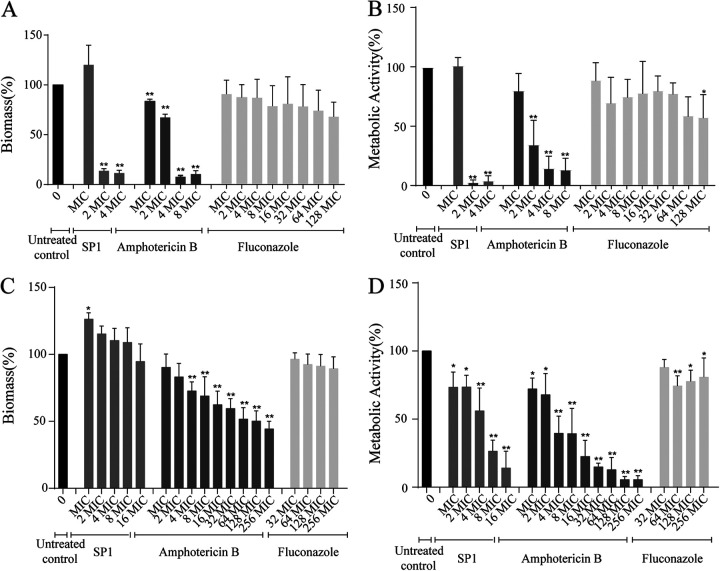
(A to D) Activity of SP1 against biofilm formation at 0 h (A and B) and mature biofilm at 72 h (C and D) of C. neoformans H99. (A and C) The biomass of the biofilms was analyzed using crystal violet staining. (B and D) The metabolic activity of the biofilms was measured using an XTT (tetrazolium salt) reduction assay. Error bars represent the standard deviation (SD) of triple replicates. Asterisks represent the *P* value significance (**, *P* < 0.01; *, *P* < 0.05) compared with the control. The MICs of SP1, amphotericin B, and fluconazole are 8 μM, 0.25 μg/mL, and 1 μg/mL, respectively.

GXM is a key constituent of the exopolymeric matrix of cryptococcal biofilm (41). We evaluated the effect of SP1 on growing and mature biofilms of C. neoformans H99. SP1 was added at the beginning of biofilm formation or after the biofilm reached maturity. The biomass and metabolic activity of biofilms were determined using crystal violet staining and the XTT (tetrazolium salt) reduction method, respectively. As shown in Fig. 6A and B, the biomass was reduced by 86.4%, and metabolic activity was reduced by 96.9% with the addition of 2 × MIC (16 μM) of SP1 (*P < *0.01) before biofilm formation. This suggested that SP1 could prevent C. neoformans H99 from efficiently forming a biofilm. As previously reported, we observed that amphotericin B inhibited biofilm formation but that fluconazole did not (31). For the mature biofilms, SP1 treatment did not reduce the biomass, which is different from the effect following treatment with amphotericin B (Fig. 6C). However, SP1 reduced the metabolic activity of the mature biofilm in a concentration-dependent manner (Fig. 6D). At concentrations of 8 × MIC (64 μM), SP1 decreased the metabolic activity of biofilm by 73.6% (Fig. 6D; *P < *0.01). Hence, our results suggest that SP1 shows strong activity against cryptococcal biofilms.

### SP1 promotes the phagocytosis of C. neoformans H99 by macrophages.

**FIG 7:**
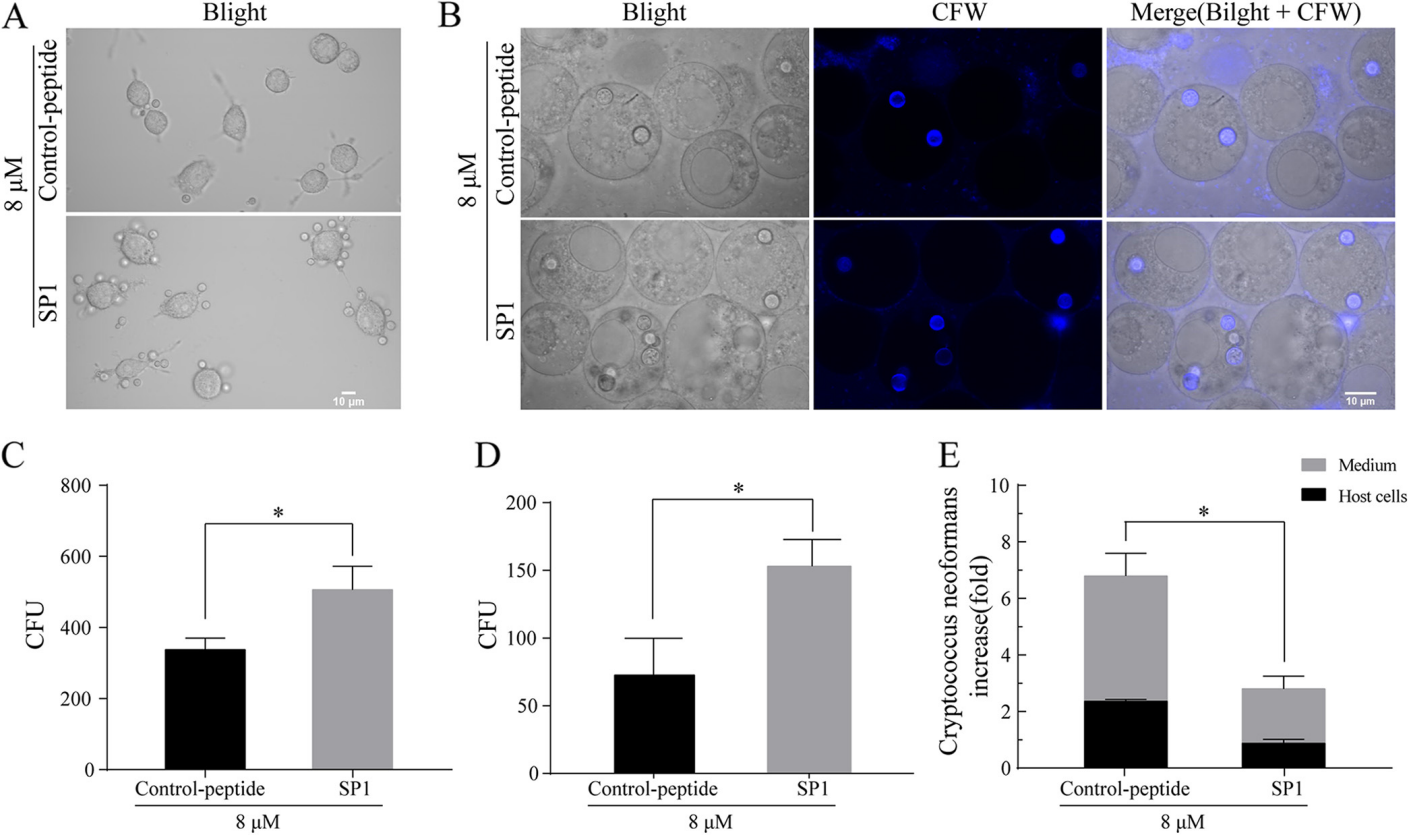
Phagocytosis of SP1-treated C. neoformans H99 cells by J774A.1 macrophages. (A) Confocal microscopic image of macrophage J774A.1 adhesion to C. neoformans H99 cells. (B) Fluorescence confocal microscopic images of phagocytosis of C. neoformans H99 cells by J774A.1 macrophages. C. neoformans H99 cells within macrophages were stained with calcofluor white (CFW). (C) Statistics of C. neoformans H99 cells adherent to macrophages. (D) Statistical analysis of the number of C. neoformans H99 cells phagocytosed by macrophages. (E) Fold changes of intracellular C. neoformans H99 cell replication. Error bars represent the standard deviation (SD) of triple replicates. Asterisks represent the *P* value significance (*, *P* < 0.05) compared with the control. C. neoformans H99 in the control group was treated with control peptide.

The polysaccharide capsule of C. neoformans is known to inhibit macrophage phagocytosis of C. neoformans (42, 43). Because SP1 damages the polysaccharide capsule, we examined whether the treatment of SP1 enhanced the phagocytosis of C. neoformans. The adhesion and phagocytosis of macrophages were assessed for SP1-pretreated C. neoformans H99 cells. The adhesion of macrophage J774A.1 to C. neoformans H99 cells in the SP1-treated group was significantly enhanced compared with that of the control group (Fig. 7A and C; *P < *0.05), and the adhesive efficiency was increased by 49.7% (Fig. 7C). After treating C. neoformans H99 cells with SP1, the number of cells phagocytosed by macrophage was nearly 2-fold that in the control group (Fig. 7B and D).

As C. neoformans can proliferate within macrophages owing to the protection of the polysaccharide capsule (18), we examined the proliferation of SP1-pretreated C. neoformans H99 that were actively ingested in macrophages. The C. neoformans H99 cells with an intact capsule or damaged capsule induced by 8 μM SP1 for 30 min were phagocytosed by J774A.1 macrophages, and fluconazole was added to inhibit the growth of C. neoformans that escaped to outside the macrophages. After 12 h from phagocytosis, the C. neoformans H99 cells with intact capsules in the control group displayed an average intracellular replication rate of 6.795-fold. However, the intracellular replication rate of SP1-pretreated cells in macrophages was only 2.805-fold (Fig. 7E; *P < *0.05). These data implied that the destruction of polysaccharide capsule by SP1 facilitated the killing by macrophages and prevented the immune escape of C. neoformans H99.

## DISCUSSION

The options for anticryptococcosis treatment are limited because only three major classes of drugs have been approved for clinical use. The situation is deteriorating because the extraordinary genomic plasticity and physiological adaptability of C. neoformans has enabled the development of extensive drug resistance (11). SP1 is a peptide that shows activity against Cryptococcus spp. and can disrupt the integrity and reduce the thickness of Cryptococcus polysaccharide capsule. In this study, we investigated the mechanism whereby SP1 damages the structure of capsule. SP1 interaction with GXM may cause the destruction of capsule. Owing to capsule damage, the treatment of SP1 produced flocculation, inhibited the formation of Cryptococcus biofilm, and enhanced the clearance of C. neoformans by macrophages. This study laid a foundation for the potential of SP1 to treat cryptococcosis.

The polysaccharide capsule is one of the primary virulence factors in C. neoformans and can enhance pathogenicity, modulate immune responses, and protect against oxidative stress (15, 44). Animal model studies suggest that the capsular mutants display reduced virulence or even avirulence (29, 45–49). The ability of SP1 to damage the capsule is a considerable advantage for the potential application in clinics. Our results demonstrated that SP1 caused the flocculation of C. neoformans H99, which may be the outcome of capsule disruption. The flocculation decreased the virulence because the adhesion property of aggregated cells enhanced the phagocytosis and clearance by lung macrophages (34). We also found that SP1 treatment enhanced the adhesion and phagocytosis of C. neoformans H99 by macrophages and hampered the replication of C. neoformans H99 within macrophages. Moreover, the obstruction of GXM release by SP1 alleviated the inhibitory effect of GXM on host immune regulation. Accordingly, SP1 not only directly killed C. neoformans but also benefited the host immune system to clear C. neoformans during the infection. In addition, SP1 would be a good candidate for drug combinations against C. neoformans, as polysaccharide capsule notoriously blocks the absorption of antifungal drugs. Studies reported that the capsule protects C. neoformans against polyenes and glycolipid-hydrolyzed inhibitors (50, 51). SP1 severely disrupts the structure of the polysaccharide capsule, which would facilitate the entry of antifungal drugs into the cells to exert fungicidal effects. The combination of SP1 and other antifungal agents would be a feasible strategy to improve therapeutic efficacy, lower side effects, and minimize development of drug resistance.

Cryptococcal biofilm has been reported to reduce the sensitivity to antifungal agents and various antimicrobial molecules produced by the innate immune system (52). GXM plays a key role in the biofilm formation of C. neoformans (30). We evaluated the effects of SP1 on biofilm formation and maturation using fluconazole and amphotericin B as the negative and positive controls, respectively. Consistent with the previous report, fluconazole showed no effect on the formation and metabolic activities of cryptococcal biofilms (31). In contrast, amphotericin B prevented the formation of cryptococcal biofilms at >0.5 μg/mL (2 × MIC) and reduced the metabolic activity of mature biofilms by at least 50% at >1 μg/mL (4 × MIC). The formation of the cryptococcal biofilm was efficiently prevented at 53.3 μg/mL (2 × MIC) SP1. Thus, SP1 prevents the release of GXM to inhibit the formation of biofilm in C. neoformans. SP1 reduced the metabolic activity of mature biofilms by at least 50% at 213.3 μg/mL (8 × MIC). Although the killing concentration required for SP1 to act on biofilms was higher than that in planktonic cells (8 × versus 1 × MIC), this is much lower than the estimated concentration (100 to 1,000 × MIC) of antifungal agents according to previous studies (53, 54). Thus, SP1 could be used to diminish the formation of cryptococcal biofilm.

GXM and GXMGal are two main polysaccharides in the C. neoformans capsule, and GXM accounts for approximately 90% of the capsule mass (55). Our study determined that SP1 directly interacted with GXM, and that *O*-acetyl modification played an important role during the interaction. The *O*-acetyl modification does not change the helical structure of GXM decasaccharide but exposes more polar groups of the GXM decasaccharide (56). Our molecular docking assay implied that the polar amino acids are important for the formation of hydrogen bonds. In addition, the isoelectric point of SP1 is 11.83, and SP1 is positively charged under neutral pH. Studies have shown that capsular polysaccharides have a negative charge. Therefore, SP1 could also interact with the polysaccharide capsule through electrostatic interaction. Although it is unclear how SP1 disrupts the polysaccharide capsule after binding to GXM, it is reasonable to postulate that SP1 disrupted the structure of the polysaccharide capsule by cross-linking the polysaccharide with SP1. Moreover, SP1 caused the flocculation of C. neoformans, and the treatment of proteinase K dispersed the flocculation. Thus, SP1 may bind to GXM and further disrupt the structure of the capsule by the aggregation of SP1. Future studies will seek to clarify the detailed molecular mechanism of how SP1 disrupts the capsule.

In conclusion, we demonstrated that SP1 interacts with GXM in the polysaccharide capsule of C. neoformans H99. The *O*-acetyl modification of GXM dramatically reinforces the interaction between SP1 and GXM. This interaction possibly leads to the disruption of the polysaccharide capsule. SP1 treatment reduces the release of GXM to the supernatant of liquid culture of C. neoformans H99. The capsule damage caused by SP1 enhances the adhesion and phagocytosis of C. neoformans H99 and impedes the proliferation of C. neoformans H99 in macrophages. These characteristics of SP1 enhance the innate immune response against the infection of C. neoformans. Furthermore, SP1 inhibits the formation of cryptococcal biofilms. As SP1 specifically kills Cryptococcus, the present findings highlight the additional benefits of SP1 for fighting against refractory C. neoformans infection by impairing C. neoformans capsule. Future studies will focus on advancing the promising clinical application of SP1.

## MATERIALS AND METHODS

### Strains, cells, and growth conditions.

C. neoformans strain H99 and H99 mutant strains (*cap59*Δ [CNAG_00721], *cas1*Δ [CNAG_07937], *uxs1*Δ [CNAG_03322], *uge1*Δ [CNAG_00697], *uge1*Δ [CNAG_03096]) were kindly provided by Linqi Wang at the Institute of Microbiology, Chinese Academy of Sciences. C. neoformans strains were revived on YPD (yeast extract peptone dextrose) solid medium at 30°C and grown in RPMI 1640 medium supplemented with MOPS (morpholinepropanesulfonic acid; 0.165 M, pH 7.0) at 37°C unless otherwise specified. Escherichia coli DH5α was cultured in LB medium at 37°C. Saccharomyces cerevisiae BY4741 was cultured in YPD medium at 30°C. Mouse macrophage cell line J774A.1 was acquired from Cuihua Liu at the Institute of Microbiology, Chinese Academy of Sciences. J774A.1 cells were maintained in Dulbecco’s modified Eagle medium (DMEM) supplemented with 10% fetal bovine serum (FBS) and cultured at 37°C with 5% CO_2_.

### Peptides and drugs.

The peptide SP1 (IRIAINGFGRIGRLVLRLALQRKDIEVVA), control peptide (IRIAINGFGRIGRPPPRPPPQRKDIEVVA), and FITC-labeled SP1 were synthesized by GenScript (Nanjing, China) as previously described (32). Fluconazole (FLC) and amphotericin B (AMB) were purchased from Meilunbio (Dalian, China).

### Flocculation experiment.

C. neoformans H99, E. coli DH5α, and S. cerevisiae BY4741 in logarithmic phase (optical density at 600 nm [OD_600_], 1) were treated with different concentrations of SP1 (0, 4, 8, 16, 32, 64, 128, 256 μM). The time required for the appearance of flocculation was recorded. Acapsular mutant *cap59*Δ in logarithmic phase (OD_600_, 1) was treated with 0 or 8 μM SP1 for 30 min. In all samples, cell-cell aggregation was checked by confocal microscopy (Olympus IX81, Japan) before and after SP1 was added. All experiments were repeated at least three times, with similar results.

### Competitive inhibition experiment.

A total of 5 mM EDTA (Sigma), 20 mM mannose (Hushi), 20 mM maltose (Hushi), 20 mM galactose (Hushi), and 20 mM glucose (Hushi) were separately added to the H99 cultures. Then 8 μM SP1 was added, and all samples were incubated at 37°C and 220 rpm for 30 min. Flocculation was determined microscopically. H99 without any treatment was the negative control, and H99 treated only with SP1 was the positive control. The probability of flocculation in 100 visual fields was counted. All experiments were repeated at least three times.

### Proteinase K treatment experiment.

After the H99 culture was treated with 8 μM SP1 for 30 min or not, it was washed three times with phosphate-buffered saline (PBS) and resuspended in an equal volume of fresh RPMI 1640 medium (supplemented with 0.165 M MOPS). Then cells were incubated with 3 mg/mL proteinase K (Amresco) at 37°C and 220 rpm for another 30 min. The results were evaluated by confocal microscopy (Olympus IX81, Japan). All experiments were repeated at least three times, with similar results.

### MIC assay.

The MIC was measured according to the previously described Clinical and Laboratory Standards Institute (CLSI) M27-A4 protocol (57). To determine the MIC of the C. neoformans strains (H99, *cas1*Δ [CNAG_07937], *uxs1*Δ [CNAG_03322], *uge1*Δ [CNAG_00697], *uge1*Δ [CNAG_03096]), they were incubated at 37°C for 72 h after the addition of SP1. RPMI 1640 (0.165 M MOPS) medium was used in the MIC assay. The value of the MIC was defined as the lowest concentration of drug without C. neoformans H99 growth. All MIC experiments were performed in triplicate.

### Indirect immunofluorescence microscopy.

According to the previous indirect immunofluorescence protocol, the polysaccharide capsule of H99 treated with 8 μM SP1 or control peptide was labeled with the capsular monoclonal antibody (MAb) 18B7 (Millipore) (58, 59). In brief, the SP1-treated or control-peptide-treated H99 culture was washed three times with PBS and resuspended in 200 μL PBS containing 20 μg/mL MAb 18B7 at a concentration of 10^7^ cells/mL. The samples were incubated at room temperature for 20 rpm for 1 h. After being washed in PBS, the samples were finally incubated with a goat anti-mouse IgG conjugate (H+L) Qdot 655 secondary antibody (Life Technologies) under the same conditions. All samples were washed again with PBS to remove unbound secondary antibodies and then examined by confocal microscopy.

### The combination of SP1 and GXM was detected by MST.

GXM was extracted and purified according to the previous experimental protocol with slight modifications (60, 61). We cultured H99 using capsule induction medium (minimal medium: 20 mg/mL thiamine, 30 mM glucose, 26 mM glycine, 20 mM MgSO_4_ · 7H_2_O, and 58.8 mM KH_2_PO_4_; pH 7; Sigma) and then extracted GXM by ethanol precipitation of polysaccharides and hexadecyltrimethylammonium bromide (CTAB) precipitation of GXM. The GXM solution was dialyzed (dialysis tube, molecular weight cutoff of 3,500) versus sterile distilled water and then lyophilized. Then GXM was weighed, aliquoted, and stored at −80°C for later use. After dissolving a part of the GXM, the GXM solution was adjusted to pH 11.25 with concentrated NH_4_OH and then incubated at 23°C for 24 h. The samples were dialyzed and lyophilized to obtain de-*O*-acetylated derivative of GXM.

The interactions between SP1 and GXM or de-*O*-acetylated GXM were determined by microscale thermophoresis (MST) assay (62). GXM (2 mg/mL), or de-*O*-acetylated GXM (2 mg/mL) was mixed with FITC-labeled SP1 after a 2-fold serial dilution. After the mixture was uniform, the solution was loaded into 16 capillaries in a gradient of concentration from high to low and placed in the MST Monolith NT.115 instrument (NanoTemper Technologies, Munich, Germany) to measure the interaction.

### Molecular modeling.

The structure of SP1 was predicted using AlphaFold2 (https://colab.research.google.com/github/sokrypton/ColabFold/blob/main/AlphaFold2.ipynb), and the structure of GXM decasaccharide with or without oxyacetylation was predicted using the GLYCAM carbohydrate builder (http://glycam.org). AutoDock Vina and PDBePISA (https://www.ebi.ac.uk/msd-srv/prot_int/pistart.html) were used for molecular docking studies. PyMOL was used to visualize the docking results and figure creation (Fig. 4C).

### Indian ink staining and capsule thickness measurement.

C. neoformans H99 was cultured to the logarithmic phase. Subsequently, 8 μM SP1 was added to the above-mentioned H99 cultures (OD_600_, 1) and then incubated at 37°C and 220 rpm for 30 min. After centrifugation, C. neoformans solution and Indian ink were mixed 1:1 and visualized with confocal microscopy (Olympus IX81, Japan). H99 not treated with SP1 was used as the control. The capsule size of 100 cells was measured using Image J software. Capsule size was defined as the width of the white part from the cell wall to the outer edge of the cell.

### Measuring the change of GXM content in the culture supernatant of C. neoformans H99.

H99 was cultured to the logarithmic phase (OD_600_, 1), and the supernatant and fungal cells were collected. The collected fungal cells were resuspended with the collected supernatant at a concentration of 2 × 10^7^ cell/mL. In the meantime, the collected supernatant was used to prepare a stock solution containing 2 × 128 μM SP1, and used to perform 2-fold gradient dilution of the stock solution. The cell suspension and different concentrations of SP1 (2 × 8, 2 × 16, 2 × 32, 2 × 64, and 2 × 128 μM) solution were mixed 1:1. Immediately after mixture, the mixed solution was centrifuged at 5,000 × *g* for 2 min, and 20 μL of the supernatant was taken to measure the content of GXM. The GXM content at this time was defined as the initial content in the supernatant before the treatment of H99 with SP1. The centrifuged mixtures were vortexed to mix them evenly and incubated at 37°C for 30 min. Subsequently, the mixtures were centrifuged, and 20 μL of the supernatant was taken to measure the content of GXM. The GXM content at this time was defined as the content in the supernatant after treatment of H99 with SP1. The content of GXM in all samples was measured using the C. neoformans capsular polysaccharide ELISA kit (Beijing Hua Bo Deyi Biotechnology Co., China) according to the instructions.

The formula for the GXM content change in the supernatant of H99 treated with different concentrations of SP1 is as follows: change of GXM content in supernatant (pg/mL) = GXM content in supernatant after SP1 treatment of H99 (pg/mL) – GXM content in supernatant before SP1 treatment of H99 (pg/mL).

### Real-time PCR of capsule-related genes.

H99 was treated with 8 μM SP1 or control peptide and then incubated at 37°C for 1 h. RNA was extracted and reverse transcribed into cDNA using the yeast RNA extraction kit (Omega) and the cDNA synthesis kit (Biotechrabbit), respectively according to the manufacturers’ instructions. The synthesized cDNA was diluted 10-fold with diethyl pyrocarbonate (DEPC) water and stored at −20°C.

*CAP10*, *MAN1*, *CMT1*, and *GRASP1* were selected for real-time PCR, and these genes are involved in polysaccharide capsular synthesis or transport (48, 63–65). GAPDH was used as the reference gene, and its quantitative PCR (qPCR) primers were as follows: 5′-TGAGAAGGACCCTGCCAACA-3′ (forward) and 5′-ACTCCGGCTTGTAGGCATCAA-3′ (reverse). The real-time PCR primers for *CAP10*, *MAN1*, *CMT1*, and *GRASP1* were derived from the research of Lee et al. (66). qPCR of H99 cDNA was performed using Hieff qPCR SYBR green master mix (Yeasen) according to the manufacturer’s instructions. Target gene expression was measured using expression relative to the GAPDH reference gene. The relative expression was calculated using the 2^–ΔΔ^*^CT^* method. All experiments were performed in triplicate.

### Antibiofilm activity.

The biofilm formation of C. neoformans H99 and the antibiofilm activity of SP1 were evaluated according to the previously described method with slight modifications (67, 68). Amphotericin B (AMB) and fluconazole (FLC) were used as biofilm inhibition and noninhibition controls, respectively, in all experiments. Here, H99 was cultured with RPMI 1640 to form biofilm. Drugs were added at 0 h and 72 h at the beginning of the culture of cryptococcal biofilm to assess the effect of the drug on biofilm formation and mature biofilm, respectively. At 0 h, SP1 at a concentration of 8 to 32 μM, AMB at a concentration of 0.25 to 2 μg/mL, and FLC at a concentration of 1 to 128 μg/mL were added and incubated at 37°C for 72 h. At 72 h, SP1 at a concentration of 8 to 128 μM, AMB at a concentration of 0.25 to 64 μg/mL, and FLC at a concentration of 32 to 256 μg/mL were added and incubated at 37°C for 48 h. The metabolic activity and total biomass of the biofilms were evaluated using the 2,3-bis(2-methoxy-4-nitro-5-sulfophenyl)-5-[(phenylamino)carbonyl]-2H-tetrazolium hydroxide (XTT) reduction assay and crystal violet staining, respectively (67). Here, the OD_492_ (for the XTT assay) and OD_590_ (for the crystal violet assay) were determined in a microplate reader (BioTek, USA). The percentage of total biomass (metabolic activity) of cryptococcal biofilm treated with SP1 (AMB/FLC) was compared to that of the untreated control. Each sample was evaluated in triplicate in three independent experiments.

### Adherence and phagocytosis assays.

The adhesion and phagocytosis assay of macrophages J774A.1 to H99 was performed according to the previously described method with slight modifications (34, 69). Here, H99 was treated with SP1 for 30 min and then washed three times with PBS and resuspended in DMEM (with 10% FBS). We treated H99 with the control peptide in the same manner. Then, SP1-treated and control-peptide-treated H99 (3 × 10^6^ cells/well) infected J774A.1 cells, respectively. After incubating C. neoformans with macrophages for 90 min and 180 min, the adhesion and phagocytosis of macrophages to C. neoformans were evaluated by determining the CFU and imaged by confocal microscopy (Olympus IX81, Japan). C. neoformans within macrophages was stained with calcofluor white (CFW; Sigma). CFW specifically stains chitin in the cell walls of several eukaryotic microorganisms, including C. neoformans (70).

### Fluconazole protection assays.

The experiment was carried out according to the previous experiment protocol (69, 71). In short, after SP1-treated or control-peptide-treated H99 infected macrophages J774A.1 for 3 h, we washed these host cells with PBS 8 to 10 times and then added fresh DMEM medium containing 20 μg/mL fluconazole. Fluconazole was used to inhibit the replication of host extracellular H99 (69). After incubation for 12 h at 37°C, the H99 cells were harvested from the DMEM medium, which contained fungal cells that escaped from the J774A.1 macrophages. At the same time, the H99 cells within the J774A.1 macrophages were also collected. The numbers of H99 cells inside and outside J774A.1 were counted by CFU. The replication number of H99 cells in the host cells is the sum of the number of H99 inside and outside the cells.

### Statistical analysis.

Student's *t* test was used to compare the data for CFU (macrophage) and capsule sizes. One-way analysis of variance (ANOVA) was used to compare the data for GXM content, gene expression, and antibiofilm activity. A *P* value of <0.05 (*) or <0.01(**) was considered statistically significant.
