# Supplementary material for: Antifungal Peptide SP1 Damages Polysaccharide Capsule of Cryptococcus neoformans and Enhances Phagocytosis of Macrophages
Source: Microbiol Spectr. 2023 Mar 14;11(2):e04562-22. doi: 10.1128/spectrum.04562-22 (PMC10100895; doi:10.1128/spectrum.04562-22)
Supplement: Supplemental file 1 — Fig. S1-S6. Download spectrum.04562-22-s0001.pdf, PDF file, 0.6 MB [file spectrum.04562-22-s0001.pdf]

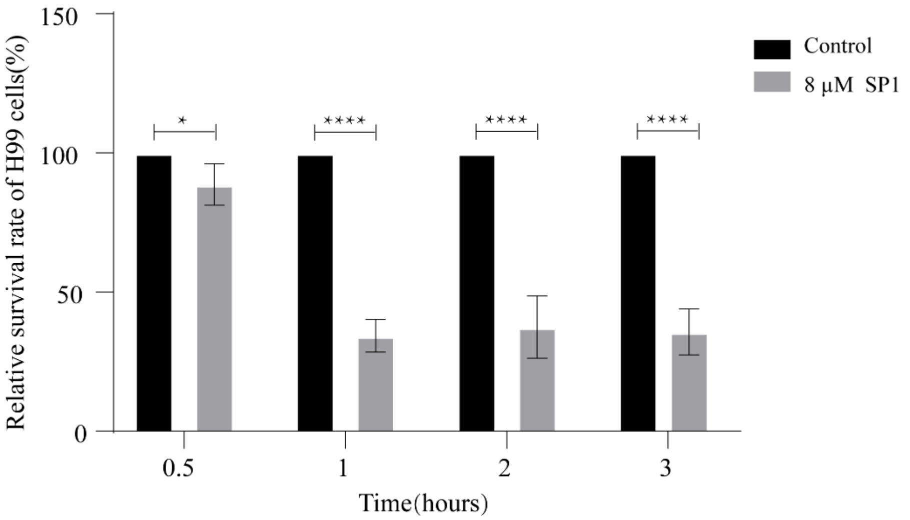

Fig S1. The survival of *C. neoformans* H99 strain after treating with 8 μM SP1 or control peptide for 0.5, 1, 2, and 3 h, respectively. The number of survival cells at the indicated time points was counted by colony-forming units(CFU). We used the relative survival rate to measure cell survival. The relative survival rate = survival rate of SP1-treated cells/survival rate of control-peptide-treated cells × 100%. Error bar represent the standard deviation of three experiments. Difference between SP1-treated and control groups was calculated using Student's t-test. * indicates $P < 0.05$, **** indicates $P < 0.0001$. The amino acid sequence of SP1 is IRIAINGFGRIGRLVLRLALQRKDIEVVA, and the amino acid sequence of control peptide is IRIAINGFGRIGRPPPRPPPQRKDIEVVA.

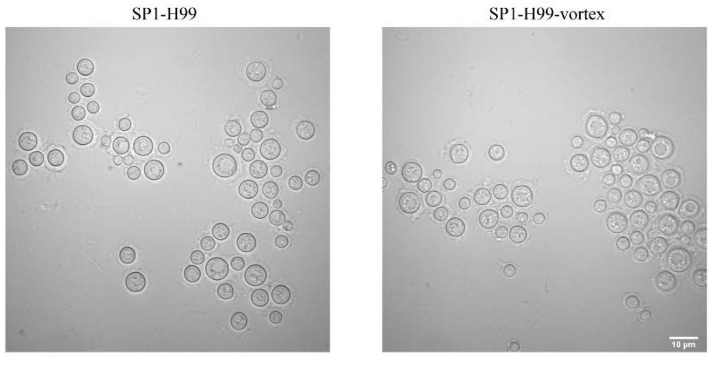

Fig S2. The cell-cell aggregation of H99 caused by SP1 is resistant to vigorous vortex.

A

Hydrogen bonds

XML

##	Structure 1	Dist. [Å]	Structure 2
1	A:ASN 6[ND2]	3.86	:0ZB 10[02]

No disulfide bonds found
No covalent bonds found
No salt bridges found

Interfacing residues (not a contact table) XML Display level: Residues

Inaccessible residues
Solvent-accessible residues
HSDC Residues making **H**ydrogen/**D**isulphide bond, **S**alt bridge or **C**ovalent link
Interfacing residues
ASA Accessible Surface Area, Å² **BSA** Buried Surface Area, Å² **Δ**

##	Structure 1	HSDC	ASA	BSA	Δ^iG
1	A:ILE 1		167.35	0.00	0.00
2	A:ARG 2		200.14	34.10	-0.02
3	A:ILE 3		126.40	0.67	0.01
4	A:ALA 4		97.89	10.57	0.14
5	A:ILE 5		159.47	11.89	-0.05
6	A:ASN 6	H	117.47	29.01	-0.12
7	A:GLY 7		50.55	0.00	0.00
8	A:PHE 8		137.73	37.01	0.59
9	A:GLY 9		47.30	0.00	0.00
10	A:ARG 10		167.84	0.00	0.00
11	A:ILE 11		121.55	0.00	0.00
12	A:GLY 12		70.08	0.00	0.00
13	A:ARG 13		165.66	0.00	0.00
14	A:LEU 14		165.45	0.00	0.00
15	A:VAL 15		107.28	0.00	0.00
16	A:LEU 16		73.45	0.00	0.00
17	A:ARG 17		171.81	0.00	0.00
18	A:LEU 18		78.99	0.00	0.00
19	A:ALA 19		113.64	0.00	0.00
20	A:LEU 20		128.34	0.00	0.00
21	A:GLN 21		132.00	0.00	0.00
22	A:ARG 22		191.11	0.00	0.00
23	A:LYS 23		86.24	0.00	0.00
24	A:ASP 24		48.82	2.57	-0.04
25	A:ILE 25		161.39	0.00	0.00
26	A:GLU 26		148.85	10.18	-0.04
27	A:VAL 27		108.32	0.00	0.00
28	A:VAL 28		92.66	0.00	0.00
29	A:ALA 29		57.31	0.00	0.00

##	Structure 2	HSDC	ASA	BSA	Δ^iG
1	:0ZB 10	H	310.76	132.09	-1.61

B

No disulfide bonds found
No covalent bonds found
No hydrogen bonds found
No salt bridges found

Interfacing residues (not a contact table) XML Display level: Residues

Inaccessible residues
Solvent-accessible residues
HSDC Residues making **H**ydrogen/**D**isulphide bond, **S**alt bridge or **C**ovalent link
Interfacing residues
ASA Accessible Surface Area, Å² **BSA** Buried Surface Area, Å² **Δ^iG** Solvation energy effect, kcal/mol |||| Buried area percentage, one bar per 10%

##	Structure 1	HSDC	ASA	BSA	Δ^iG
1	A:ILE 1		208.74	64.97	0.59
2	A:ARG 2		111.21	1.17	0.02
3	A:ILE 3		161.31	0.00	0.00
4	A:ALA 4		63.87	0.00	0.00
5	A:ILE 5		148.04	0.00	0.00
6	A:ASN 6		118.80	0.00	0.00
7	A:GLY 7		66.77	0.00	0.00
8	A:PHE 8		189.28	0.00	0.00
9	A:GLY 9		43.97	0.00	0.00
10	A:ARG 10		154.69	0.00	0.00
11	A:ILE 11		123.81	0.00	0.00
12	A:GLY 12		13.16	0.00	0.00
13	A:ARG 13		159.17	0.00	0.00
14	A:LEU 14		64.22	0.00	0.00
15	A:VAL 15		68.36	3.08	0.05
16	A:LEU 16		118.61	0.00	0.00
17	A:ARG 17		120.52	0.00	0.00
18	A:LEU 18		133.42	0.00	0.00
19	A:ALA 19		63.18	0.00	0.00
20	A:LEU 20		106.45	0.00	0.00
21	A:GLN 21		85.19	0.00	0.00
22	A:ARG 22		105.71	0.00	0.00
23	A:LYS 23		142.16	46.02	0.18
24	A:ASP 24		104.90	17.84	-0.19
25	A:ILE 25		128.74	0.00	0.00
26	A:GLU 26		143.06	0.00	0.00
27	A:VAL 27		100.62	0.00	0.00
28	A:VAL 28		114.89	0.00	0.00
29	A:ALA 29		106.78	0.00	0.00

##	Structure 2	HSDC	ASA	BSA	Δ^iG
1	:0XB 8		263.32	130.74	0.00

Fig S3. The results of molecular docking were analyzed using PDBePISA (https://www.ebi.ac.uk/msd-srv/prot_int/pistart.html). (A) The results of molecular docking of SP1 and GXM were analyzed using PDBePISA. (B) The results of molecular docking of SP1 and GXM without oxyacetylation were analyzed using PDBePISA. Amino acid interaction with $\Delta^iG < 0$ is the criteria for determining an interaction.

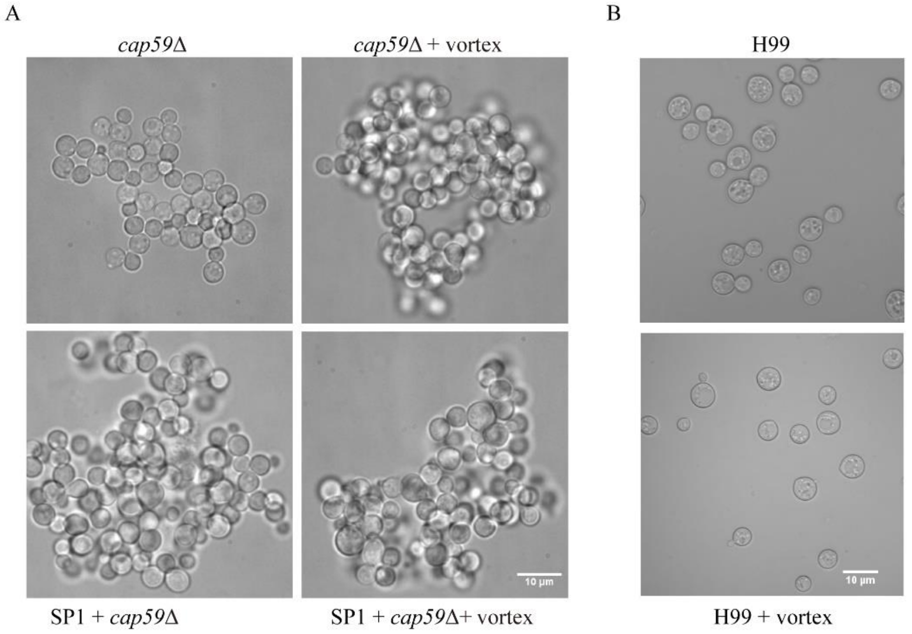

Fig S4. (A)The cell aggregation of *cap59*Δ mutant, which occurred spontaneously or with the treatment of 8 μM SP1, was resistant to vigorous vortex. (B) The transient cell aggregates of *C. neoformans* H99 can be dispersed by vortex.

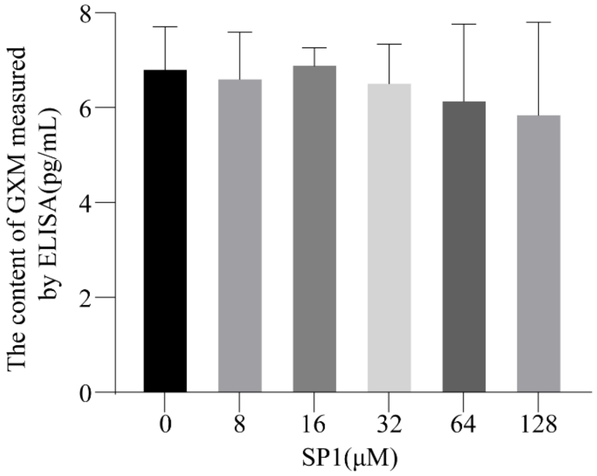

Fig S5. SP1 does not affect the measurement of GXM content by ELISA. The supernatant of *C. neoformans* H99 was collected when cells were cultured to logarithmic phase (OD_{600} = 1). The supernatant was then incubated with different concentrations of SP1(0, 4, 8, 16, 32, 64, 128 μM) for 30 min, followed ELISA to measure of the content of GXM. Error bar represent the standard deviation of three experiments. One-way analysis of variance (ANOVA) was used to analyze the significance. $P < 0.05$ indicates a significant difference.

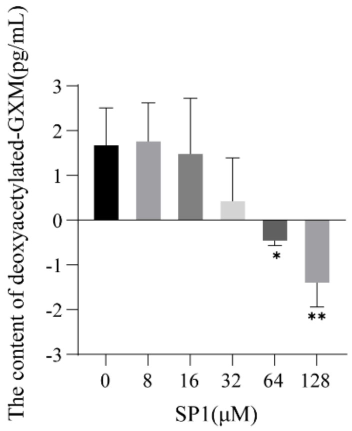

Fig S6. The content of deoxyacetylated-GXM in the supernatant after treating *C. neoformans cas1*Δ cells with SP1 for 30 minutes. The content of deoxyacetylated-GXM was measured by ELISA. Asterisks represent P-value significance (** $P < 0.01$, * $P < 0.05$) calculated using one-way analysis of variance (ANOVA).
